# RobOMP: Robust variants of Orthogonal Matching Pursuit for sparse representations

**DOI:** 10.7717/peerj-cs.192

**Published:** 2019-05-13

**Authors:** Carlos A. Loza

**Affiliations:** Department of Mathematics, Universidad San Francisco de Quito, Quito, Ecuador

**Keywords:** M-Estimation, Matching Pursuit, Representation-based classifier, Robustclassification, Sparse representation, Outliers

## Abstract

Sparse coding aims to find a parsimonious representation of an example given an observation matrix or dictionary. In this regard, Orthogonal Matching Pursuit (OMP) provides an intuitive, simple and fast approximation of the optimal solution. However, its main building block is anchored on the minimization of the Mean Squared Error cost function (MSE). This approach is only optimal if the errors are distributed according to a Gaussian distribution without samples that strongly deviate from the main mode, i.e. outliers. If such assumption is violated, the sparse code will likely be biased and performance will degrade accordingly. In this paper, we introduce five robust variants of OMP (RobOMP) fully based on the theory of M-Estimators under a linear model. The proposed framework exploits efficient Iteratively Reweighted Least Squares (IRLS) techniques to mitigate the effect of outliers and emphasize the samples corresponding to the main mode of the data. This is done adaptively via a learned weight vector that models the distribution of the data in a robust manner. Experiments on synthetic data under several noise distributions and image recognition under different combinations of occlusion and missing pixels thoroughly detail the superiority of RobOMP over MSE-based approaches and similar robust alternatives. We also introduce a denoising framework based on robust, sparse and redundant representations that open the door to potential further applications of the proposed techniques. The five different variants of RobOMP do not require parameter tuning from the user and, hence, constitute principled alternatives to OMP.

## Introduction

Sparse modeling is a learning framework with relevant applications in areas where parsimonious representations are considered advantageous, such as signal processing, machine learning, and computer vision. Dictionary learning, image denoising, image super–resolution, visual tracking and image classification constitute some of the most celebrated applications of sparse modeling (Aharon, Elad & Bruckstein, 2006; Elad & Aharon, 2006; Mallat, 2008; Wright et al., 2009; Elad, Figueiredo & Ma, 2010; Xu et al., 2011). Strictly speaking, sparse modeling refers to the entire process of designing and learning a model, while sparse coding, sparse representation, or sparse decomposition is an inference process—estimation of the latent variables of such model. The latter formally emerged as a machine learning adaptation of the sparse coding scheme found in the mammalian primary visual cortex (Olshausen & Field, 1996).

The sparse coding problem is inherently combinatorial and, therefore, intractable in practice. Thus, classic solutions involve either greedy approximations or relaxations of the original ℓ_0_-pseudonorm. Examples of the former family of algorithms include Matching Pursuit (MP) and all of its variants (Mallat & Zhang, 1993), while Basis Pursuit (Chen, Donoho & Saunders, 2001) and Lasso (Tibshirani, 1996) are the archetypes of the latter techniques. Particularly, Orthogonal Matching Pursuit (OMP) is usually regarded as more appealing due to its efficiency, convergence properties, and simple, intuitive implementation based on iterative selection of the most correlated predictor to the current residual and batch update of the entire active set (Tropp & Gilbert, 2007).

The success of OMP is confirmed by the many variants proposed in the literature. Wang, Kwon & Shim (2012) introduced Generalized OMP (GOMP) where more than one predictor or atom (i.e., columns of the measurement matrix or dictionary) are selected per iteration. Regularized OMP (ROMP) exploits a predefined regularization rule (Needell & Vershynin, 2010), while CoSaMP incorporates additional pruning steps to refine the estimate recursively (Needell & Tropp, 2009). The implicit foundation of the aforementioned variants—and, hence, of the original OMP—is optimization based on Ordinary Least Squares (OLS), which is optimal under a Mean Squared Error (MSE) cost function or, equivalently, a Gaussian distribution of the errors. Any deviation from such assumptions, e.g., outliers, impulsive noise or non–Gaussian additive noise, would result in biased estimations and performance degradation in general.

Wang, Tang & Li (2017) proposed Correntropy Matching Pursuit (CMP) to mitigate the detrimental effect of outliers in the sparse coding process. Basically, the Correntropy Induced Metric replaces the MSE as the cost function of the iterative active set update of OMP. Consequently, the framework becomes robust to outliers and impulsive noise by weighing the input samples according to a Gaussian kernel. The resulting non–convex performance surface is optimized via the Half–Quadratic (HQ) technique to yield fast, iterative approximations of local optima (Geman & Yang, 1995; Nikolova & Ng, 2005). Even though the algorithm is successful in alleviating the effect of outliers in practical applications, the main hyperparameter—the Gaussian kernel bandwidth—is chosen empirically with no theoretical validation. With this mind, we propose a generalization of CMP by reformulating the active set update under the lens of robust linear regression; specifically, we exploit the well known and developed theory of M–Estimators (Andersen, 2008; Huber, 2011) to devise five different robust variants of OMP: RobOMP. Each one utilizes validated hyperparameters that guarantee robustness up to theoretical limits. In addition, the HQ optimization technique is reduced to the Iteratively Reweighted Least Squares (IRLS) algorithm in order to provide an intuitive and effective implementation while still enjoying the weighing nature introduced in CMP.

**Figure 1:**
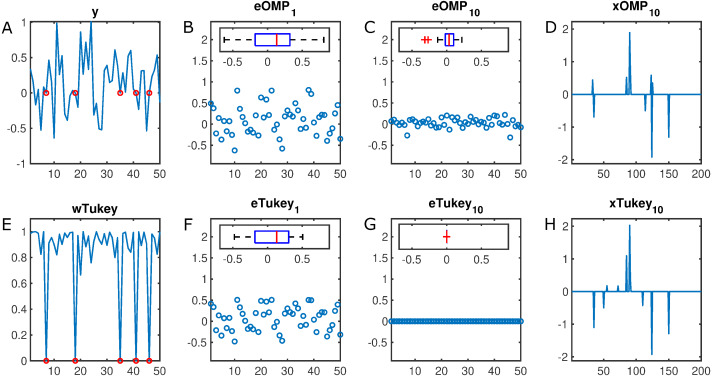
Illustration of the robustness of the proposed method. (A) *y* ∈ IR^50^ constitutes an observation vector with five missing samples (set to zero, marked in red). (B) *eOMP*_1_ and (C) *eOMP*_10_ are the resulting errors after the first and tenth iteration of OMP (with corresponding box plots as insets), respectively. (D) *xOMP*_10_ is the final estimated sparse decomposition after 10 OMP iterations. Their RobOMP counterparts (Tukey estimator) (F–G) reduce more aggressively the dynamic range of the errors until almost collapsing to a delta distribution; this results in optimal sparse coding (H). (E) *wTukey* is the learned weight vector that assigns values close to one to values around the main mode of the data and small weights to potential outliers (red marks). Number of iterative sparse decompositions equal to ground truth cardinality of sparse active set, i.e., *K* = *K*_0_ = 10.

For instance, Fig. 1 illustrates the estimated error in a 50–dimensional observation vector with a 10% rate of missing samples (set equal to zero). While Tukey–Estimator–based–OMP practically collapses the error distribution after 10 decompositions, the OMP counterpart still leaves a remnant that derives in suboptimal sparse coding. Moreover, RobOMP provides an additional output that effectively weighs the components of the input space in a [0,1] scale. In particular, the missing samples are indeed assigned weights close to zero in order to alleviate their effect in the estimation of the sparse decomposition.

We present three different sets of results to validate the proposed robust, sparse inference framework. First, synthetic data with access to ground truth (support of the representation) highlights the robustness of the estimators under several types of noise, such as additive non–Gaussian densities and instance–based degradation (e.g., missing samples and impulsive noise). Then, a robust sparse representation–based classifier (RSRC) is developed for image recognition under missing pixels and occlusion scenarios. The results outperform the OMP–based variants and the CMP–based classifier (CMPC) for several cases. Lastly, preliminary results on image denoising via sparse and redundant representations over overcomplete dictionaries are presented with the hope of exploiting RobOMP in the future for image denoising under non–Gaussian additive noise. The rest of the paper is organized as follows: Section 2 details the state of the art and related work concerning greedy approximations to the sparse coding problem. Section 3 introduces the theory, rationale, and algorithms regarding M–estimation–based Robust OMP: RobOMP. Section 4 details the results using synthetic data and popular digital image databases, while Section 5 discusses more in–depth technical concepts, analyzes the implications of the proposed framework, and offers potential further work. Lastly, Section 6 concludes the paper.

## State of the Art and Related Work

Let **y** ∈ IR^*m*^ be a measurement vector with an ideal, noiseless, sparse representation, **x**_0_ ∈ IR^*n*^, with respect to the measurement matrix (also known as dictionary), **D** ∈ IR^*m*×*n*^. The matrix **D** is usually overcomplete, i.e., *m* < *n*, to promote sparse decompositions. In practice, **y** is affected by a noise component, **n** ∈ IR^*m*^. This results in the following constrained, linear, additive model:

(1) \begin{eqnarray*}\mathbf{y}={\mathbf{Dx}}_{0}+\mathbf{n} \text{s.t.} {|}{|}{\mathbf{x}}_{0}{|}{{|}}_{0}={K}_{0}\end{eqnarray*}

where *K*_0_ indicates the support of the sparse decomposition and ||⋅||_0_ represents the ℓ_0_–pseudonorm, i.e., number of non–zero components in **x**_0_. The sparse coding framework aims to estimate **x**_0_ given the measurement vector and matrix plus a sparsity constraint.

### MSE–based OMP

Orthogonal Matching Pursuit (Tropp & Gilbert, 2007) attempts to find the locally optimal solution by iteratively estimating the most correlated atom in **D** to the current residual. In particular, OMP initializes the residual **r**_0_ = **y**, the set containing the indices of the atoms that are part of the decomposition (an active set) Λ_0_ = ∅, and the iteration *k* = 1. In the *k*th iteration, the algorithm finds the predictor most correlated to the current residual:

(2) \begin{eqnarray*}{\lambda }_{k}={argmax}_{i\in \Omega }{|}\langle {\mathbf{r}}_{k-1},{\mathbf{d}}_{i}\rangle {|}\end{eqnarray*}

where 〈⋅, ⋅〉 denotes the inner product operator, **d**_*i*_ represents the *i*th column of **D**, and Ω = {1, 2, …, *n*}. The resulting atom is added to the active set via Λ, i.e.:

(3) \begin{eqnarray*}{\Lambda }_{k}={\Lambda }_{k-1}\cup {\lambda }_{k}.\end{eqnarray*}

The next step is the major refinement of the original Matching Pursuit algorithm (Mallat & Zhang, 1993)—instead of updating the sparse decomposition one component at the time, OMP updates all the coefficients corresponding to the active set at once according to a MSE criterion

(4) \begin{eqnarray*}{\mathbf{x}}_{k}=\argmin _{\mathbf{x}\in {\mathrm{IR}}^{n},\text{supp}(\mathbf{x})\subset {\Lambda }_{k}}{|}{|}\mathbf{y}-\mathbf{Dx}{|}{{|}}_{2}\end{eqnarray*}

where supp (**x**) is the support set of vector **x**. Equation (4) can be readily solved via OLS or Linear Regression where the predictors are the columns of **D** indexed by Λ_*k*_ and the response is the measurement vector **y**. Stopping criterion for OMP typically include a set number of iterations or compliance with a set minimum error of the residue. In the end, the estimated sparse code, **x**, is set as the last **x**_*k*_ obtained.

In practice, the true sparsity pattern, *K*_0_, is unknown and the total number of OMP iterations, *K*, is treated as a hyperparameter. For a detailed analysis regarding convergence and recovery error bounds of OMP, see Donoho, Elad & Temlyakov (2006). A potential drawback of OMP is the extra computational complexity added by the OLS solver. Specifically, each incremental update of the active set affects the time complexity of the algorithm in a polynomial fashion: $\mathcal{O}({k}^{2}n+{k}^{3})$ where *k* is the current iteration.

Generalized Orthogonal Matching Pursuit (Wang, Kwon & Shim, 2012) refines OMP by selecting *N*_0_ atoms per iteration. If the indices of the active set columns in the *k*th iteration are denoted as *J*_*k*_[1], *J*_*k*_[2], …, *J*_*k*_[*N*_0_], then *J*_*k*_[*j*] can be defined recursively:

(5) \begin{eqnarray*}{J}_{k}[j]={argmax}_{i\in \Omega \setminus \{{J}_{k}[1],\ldots ,{J}_{k}[j-1]\}}{|}\langle {\mathbf{r}}_{k-1},{\mathbf{d}}_{i}\rangle {|}, 1\leq j\leq {N}_{0}\end{eqnarray*}

The index set ${\{{J}_{k}[j]\}}_{j=1}^{{N}_{0}}$ is then added to Λ_*k*_ and, likewise OMP, GOMP exploits an OLS solver to update the current active set. Both OMP and GOMP obtain locally optimal solutions under the assumption of Gaussianity (or Normality) of the errors. Yet, if such restriction is violated (e.g., by the presence of outliers), the estimated sparse code, **x**, will most likely be biased.

### CMP

The main drawback of MSE–based cost functions is its weighing nature in terms of influence and importance assigned to the available samples. In particular, MSE considers every sample as equally important and assigns a constant weight equal to one to all the inputs. Wang, Tang & Li (2017) proposed exploiting Correntropy (Liu, Pokharel & Príncipe, 2007) instead of MSE as an alternative cost function in the greedy sparse coding framework. Basically, the novel loss function utilizes the Correntropy Induced Metric (CIM) to weigh samples according to a Gaussian kernel *g*_*σ*_(*t*) = exp(−*t*^2^∕2*σ*^2^), where *σ*, the kernel bandwidth, modulates the norm the CIM will mimic, e.g., for small *σ*, the CIM behaves similar to the ℓ_0_-pseudonorm (aggressive non–linear weighing), if *σ* increases, CIM will mimic the ℓ_1_–norm (moderate linear weighing), and, lastly, for large *σ*, the resulting cost function defaults to MSE, i.e., constant weighing for all inputs. The main conclusion here is that the CIM, unlike MSE, is robust to outliers for a principled choice of *σ*. This outcome easily generalizes for non–Gaussian environments with long–tailed distributions on the errors.

Correntropy Matching Pursuit (CMP) exploits the CIM robustness to update the active set in the sparse coding solver. The algorithm begins in a similar fashion as OMP, i.e., **r**_0_ = **y**, Λ_0_ = ∅, and *k* = 1. Then, instead of the MSE–based update of Eq. (4), CMP proceeds to minimize the following CIM–based expression:

(6) \begin{eqnarray*}{\mathbf{x}}_{k}=\argmin _{\mathbf{x}\in {\mathrm{IR}}^{n},\text{supp}(\mathbf{x})\subset {\Lambda }_{k}}{L}_{\sigma }(\mathbf{y}-\mathbf{Dx})\end{eqnarray*}

where ${L}_{\sigma }(\mathbf{e})= \frac{1}{m} {\mathop{\sum }\nolimits }_{i=1}^{m}{\sigma }^{2}(1-{g}_{\sigma }(\mathbf{e}[i]))$ is the simplified version (without constant terms independent of **e**) of the CIM loss function and **e**[*i*] is the *i*th entry of the vector **e**. The Half–Quadratic (HQ) technique is utilized to efficiently optimize the invex CIM cost function (Geman & Yang, 1995; Nikolova & Ng, 2005). The result is a local minimum of Eq. (6) alongside a weight vector **w** that indicates the importance of the components of the observation vector **y**:

(7) \begin{eqnarray*}{\mathbf{w}}^{(t+1)}[i]={g}_{\sigma }(\mathbf{y}[i]-({\mathbf{D}\mathbf{x}}^{(t)})[i]), i=1,2,\ldots ,m\end{eqnarray*}

where *t* is the iteration in the HQ subroutine. In short, the HQ optimization performs block coordinate descent to separately optimize the sparse code, **x**, and the weight vector, **w**, in order to find local optima. The hyperparameter *σ* is iteratively updated without manual selection according to the following heuristic:

(8) \begin{eqnarray*}{\sigma }^{(t+1)}={ \left( \frac{1}{2m} {\mathop{ \left\| \right. \mathbf{y}-{\mathbf{D}\mathbf{x}}^{(t+1)} \left\| \right. }\nolimits }_{2}^{2} \right) }^{1/2}.\end{eqnarray*}

In Wang, Tang & Li (2017), the authors thoroughly illustrate the advantage of CMP over many MSE–based variants of OMP when dealing with non-Gaussian error distributions and outliers in computer vision applications. And even though they mention the improved performance of the algorithm when *σ* is iteratively updated versus manual selection scenarios, they fail to explain the particular heuristic behind Eq. (8) or its statistical validity. In addition, the HQ optimization technique is succinctly reduced to a weighted Least Squares problem than can be solved explicitly. Therefore, more principled techniques that exploit weighted Least Squares and robust estimators for linear regression can readily provide the needed statistical validity, while at the same time, generalize the concepts of CMP under the umbrella of M–estimators.

## Robust Orthogonal Matching Pursuit

MSE–based OMP appeals to OLS solvers to optimize Eq. (4). In particular, let Φ ∈ IR^*m*×*k*^ correspond to the *active* atoms in the dictionary **D** at iteration *k*, i.e., Φ = [**d**_Λ_*k*_[1]_, **d**_Λ_*k*_[2]_, …, **d**_Λ_*k*_[*k*]_], and *β* ∈ IR^*k*^ be the vector corresponding to the coefficients that solve the following regression problem:

(9) \begin{eqnarray*}\mathbf{y}=\Phi \beta +\mathbf{e}\end{eqnarray*}

where **e** is an error vector with independent components identically distributed according to a zero–mean Normal density ($\mathbf{e}[i]&sim; \mathcal{N}(0,{\mathrm{&sigma;}}^{2})$). Then, the least squares regression estimator, $\hat {\beta }$, is the maximum likelihood estimator for *β* under a Gaussian density prior, i.e.:

(10) \begin{eqnarray*}\hat {\beta }={argmax}_{\beta }\prod _{i=1}^{m} \frac{1}{\sqrt{2\pi {\sigma }^{2}}} \exp \nolimits \left( \right. - \frac{\mathbf{e}[i]^{2}}{2{\sigma }^{2}} \left( \right. ={argmax}_{\beta }\prod _{i=1}^{m} \frac{1}{\sqrt{2\pi {\sigma }^{2}}} \exp \nolimits \left( \right. - \frac{(\mathbf{y}[i]-(\Phi \beta )[i])^{2}}{2{\sigma }^{2}} \left( \right. \nonumber\\\displaystyle \end{eqnarray*}

which is equivalent to maximizing the logarithm of Eq. (10) over *β*:

(11) \begin{eqnarray*}\hat {\beta }={argmax}_{\beta }\sum _{i=1}^{m} \left( \right. - \frac{1}{2} \ln \nolimits (2\pi {\sigma }^{2})- \frac{\mathbf{e}[i]^{2}}{2{\sigma }^{2}} \left( \right. =\argmin _{\beta }\sum _{i=1}^{m} \left( \right. \frac{\mathbf{e}[i]^{2}}{2} \left( \right. .\end{eqnarray*}

Since *σ* is assumed as constant, $\hat {\beta }$ is the estimator that minimizes the sum of squares of the errors, or residuals. Hence, the optimal solution is derived by classic optimization theory giving rise to the well known normal equations and OLS estimator:

\begin{eqnarray*}\sum _{i=1}^{m}\mathbf{e}[i]^{2}={\mathbf{e}}^{T}\mathbf{e}\nonumber\\\displaystyle =(\mathbf{y}-\Phi \beta )^{T}(\mathbf{y}-\Phi \beta )\nonumber\\\displaystyle ={\mathbf{y}}^{T}\mathbf{y}-{\mathbf{y}}^{T}\Phi \beta -{\beta }^{T}{\Phi }^{T}\mathbf{y}+{\beta }^{T}{\Phi }^{T}\Phi \beta . \end{eqnarray*}

At the minimum:

\begin{eqnarray*} \frac{\delta }{\delta \beta } \sum _{i=1}^{m}\mathbf{e}[i]^{2}=0= \frac{\delta }{\delta \beta } ({\mathbf{y}}^{T}\mathbf{y}-{\mathbf{y}}^{T}\Phi \beta -{\beta }^{T}{\Phi }^{T}\mathbf{y}+{\beta }^{T}{\Phi }^{T}\Phi \beta )\nonumber\\\displaystyle =0-{\Phi }^{T}y-{\Phi }^{T}y+2({\Phi }^{T}\Phi )\beta . \end{eqnarray*}

Consequently when Φ^*T*^Φ is non–singular, the optimal estimated coefficients vector has a closed–form solution equal to:

(12) \begin{eqnarray*}{\hat {\beta }}_{\text{OLS}}=\hat {\beta }=({\Phi }^{T}\Phi )^{-1}{\Phi }^{T}\mathbf{y}\end{eqnarray*}

which is optimal under a Gaussian distribution of the errors. If such assumption is no longer valid due to outliers or non–Gaussian environments, M–Estimators provide a suitable alternative to the estimation problem.

### M–Estimators

If the errors are not normally distributed, the estimator of Eq. (12) will be suboptimal. Hence, a different function is utilized to model the statistical properties of the errors. Following the same premises of independence and equivalence of the optimum under the log–transform, the following estimator arises:

(13) \begin{eqnarray*}{\hat {\beta }}_{M-Est}=\argmin _{\beta }\sum _{i=1}^{m}\rho \left( \right. \frac{\mathbf{e}[i]}{s} \left( \right. =\argmin _{\beta }\sum _{i=1}^{m}\rho \left( \right. \frac{(\mathbf{y}[i]-(\Phi \beta )[i])}{s} \left( \right. \end{eqnarray*}

where *ρ*(*e*) is a continuous, symmetric function (also known as the objective function) with a unique minimum at *e* = 0 (Andersen, 2008). Clearly, *ρ*(*e*) reduces to half the sum of squared errors for the Gaussian case. *s* is an estimate of the scale of the errors in order to guarantee scale–invariance of the solution. The usual standard deviation cannot be used for *s* due to its non–robustness; thus, a suitable alternative is usually the “re–scaled MAD”:

(14) \begin{eqnarray*}s=1.4826\times MAD\end{eqnarray*}

where the *MAD* (median absolute deviation) is highly resistant to outliers with a breakdown point (BDP) of 50%, as it is based on the median of the errors ($\tilde {\mathbf{e}}$) rather than their mean (Andersen, 2008):

(15) \begin{eqnarray*}MAD=\text{median}{|}\mathbf{e}[i]-\tilde {\mathbf{e}}{|}.\end{eqnarray*}

The re–scaling factor of 1.4826 guarantees that, for large sample sizes and $\mathbf{e}[i]&sim; \mathcal{N}(0,{\mathrm{&sigma;}}^{2})$, *s* reduces to the population standard deviation (Hogg, 1979). M–Estimation then, likewise OLS, finds the optimal coefficients vector via partial differentiation of Eq. (13) with respect to each of the *k* parameters in question, resulting in a system of *k* equations:

(16) \begin{eqnarray*}\sum _{i=1}^{m}{\Phi }_{ij}\psi \left( \right. \frac{\mathbf{y}[i]-{\phi }_{i}^{T}\beta }{s} \left( \right. =\sum _{i=1}^{m}{\Phi }_{ij}\psi \left( \right. \frac{\mathbf{e}[i]}{s} \left( \right. =0, j=1,2,\ldots ,k\end{eqnarray*}

where *ϕ*_*i*_ represents the *i*th row of the matrix Φ while Φ_*ij*_ accesses the *j*th component of the *i*th row of $\Phi .\psi \left( \right. \frac{\mathbf{e}[i]}{s} \left( \right. = \frac{\partial \rho }{\partial \frac{\mathbf{e}[i]}{s} } $ is known as the score function while the weight function is derived from it as:

(17) \begin{eqnarray*}\mathbf{w}[i]=\mathbf{w} \left( \right. \frac{\mathbf{e}[i]}{s} \left( \right. = \frac{\psi \left( \right. \frac{\mathbf{e}[i]}{s} \left( \right. }{ \frac{\mathbf{e}[i]}{s} } .\end{eqnarray*}

Substituting Eqs. (17) into (16) results in:

(18) \begin{eqnarray*}\sum _{i=1}^{m}{\Phi }_{ij}\mathbf{w}[i] \frac{\mathbf{e}[i]}{s} =\sum _{i=1}^{m}{\Phi }_{ij}\mathbf{w}[i](\mathbf{y}[i]-{\phi }_{i}^{T}\beta ) \frac{1}{s} =0 j=1,2,\ldots ,k\nonumber\\\displaystyle \sum _{i=1}^{m}{\Phi }_{ij}\mathbf{w}[i](\mathbf{y}[i]-{\phi }_{i}^{T}\beta ) \frac{1}{s} =0 j=1,2,\ldots ,k\nonumber\\\displaystyle \sum _{i=1}^{m}{\Phi }_{ij}\mathbf{w}[i]{\phi }_{i}\beta =\sum _{i=1}^{m}{\Phi }_{ij}\mathbf{w}[i]\mathbf{y}[i] j=1,2,\ldots ,k\end{eqnarray*}

which can be succinctly reduced in matrix form as:

(19) \begin{eqnarray*}{\Phi }^{T}\mathbf{W}\Phi \beta ={\Phi }^{T}\mathbf{W}\mathbf{y}\end{eqnarray*}

by defining the weight matrix, **W**, as a square diagonal matrix with non–zero elements equal to the entries in **w**, i.e.: **W** = diag({**w**[*i*]:*i* = 1, 2, …, *m*}). Lastly, if Φ^*T*^**W**Φ is well-conditioned, the closed form solution of the robust M–Estimator is equal to:

(20) \begin{eqnarray*}{\hat {\beta }}_{M-Est}=({\Phi }^{T}\mathbf{W}\Phi )^{-1}{\Phi }^{T}\mathbf{Wy}.\end{eqnarray*}

Equation (20) resembles its OLS counterpart (Eq. (12)), except for the addition of the matrix **W** that weights the entries of the observation vector and mitigates the effect of outliers according to a linear fit. A wide variety of objective functions (and in turn, weight functions) have been proposed in the literature (for a thorough review, see Zhang (1997)). For the present study, we will focus on five different variants that are detailed in Table 1. Every M–Estimator weighs its entries according to a symmetric, decaying function that assigns large weights to errors in the vicinity of zero and small coefficients to gross contributions. Consequently, the estimators downplay the effect of outliers and samples, in general, that deviate from the main mode of the residuals.

**Table 1:** Comparison between OLS estimator and M–Estimators. Objective *ρ*(*e*) and weight w(e) functions of OLS solution and five different M–Estimators. For M–Estimators, error entries are standardized, i.e. divided by the scale estimator, *s*. Each robust variant comes with a hyperparameter *c*. Exemplary plots in the last column utilize the optimal hyperparameters detailed in Table 2.

Type	*ρ*(*e*)	*w*(*e*)	*w*(*e*)
OLS	$ \frac{1}{2} {e}^{2}$	1	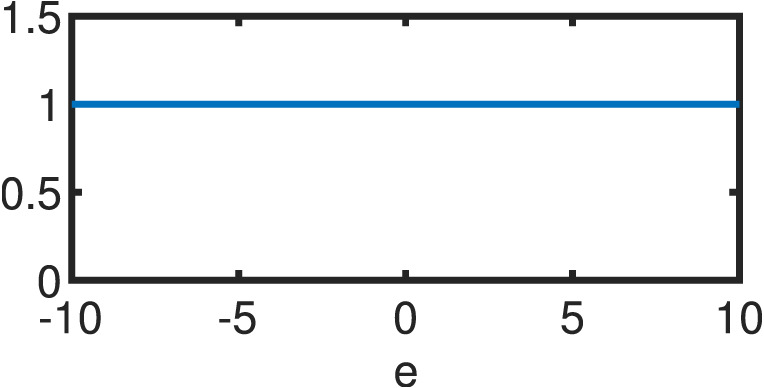
Cauchy	$ \frac{{c}^{2}}{2} \log (1+( \frac{e}{c} )^{2})$	$ \frac{1}{1+(e/c)^{2}} $	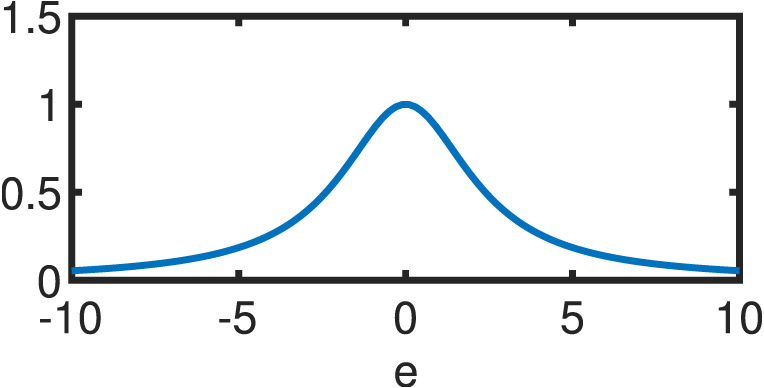
Fair	${c}^{2} \left( \right. \frac{{|}e{|}}{c} -\log \left( \right. 1+ \frac{{|}e{|}}{c} \left( \right. \left( \right. $	$ \frac{1}{1+{|}e{|}/c} $	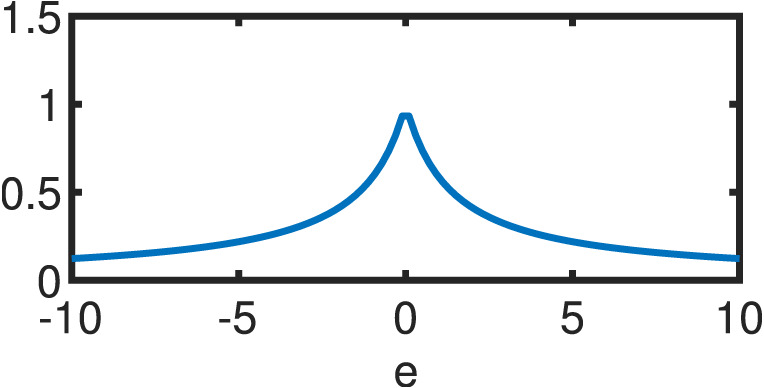
Huber \begin{eqnarray*} \left\{ \right. \begin{array}{@{}l@{}} \displaystyle \text{if}{|}e{|}\leq c\\ \displaystyle \text{if}{|}e{|}\geq c\\ \displaystyle \end{array} \end{eqnarray*}	\begin{eqnarray*} \left\{ \right. \begin{array}{@{}l@{}} \displaystyle \frac{{e}^{2}}{2} \\ \displaystyle c \left( \right. {|}e{|}- \frac{c}{2} \left( \right. \\ \displaystyle \end{array} \end{eqnarray*}	\begin{eqnarray*} \left\{ \right. \begin{array}{@{}l@{}} \displaystyle 1\\ \displaystyle \frac{c}{{|}e{|}} \\ \displaystyle \end{array} \end{eqnarray*}	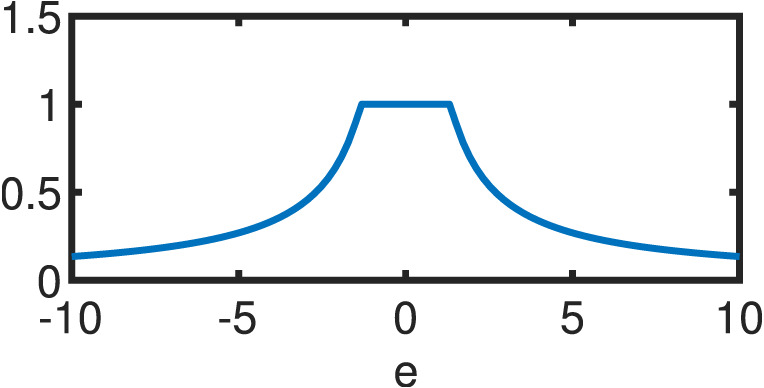
Tukey \begin{eqnarray*} \left\{ \right. \begin{array}{@{}l@{}} \displaystyle \text{if}{|}e{|}\leq c\\ \displaystyle \text{if}{|}e{|}\gt c\\ \displaystyle \end{array} \end{eqnarray*}	\begin{eqnarray*} \left\{ \right. \begin{array}{@{}l@{}} \displaystyle \frac{{c}^{2}}{6} \left( \right. 1- \left( \right. 1- \left( \right. \frac{e}{c} { \left( \right. }^{2}{ \left( \right. }^{3} \left( \right. \\ \displaystyle \frac{{c}^{2}}{6} \\ \displaystyle \end{array} \end{eqnarray*}	\begin{eqnarray*} \left\{ \right. \begin{array}{@{}l@{}} \displaystyle \left( \right. 1-( \frac{e}{c} )^{2}{ \left( \right. }^{2}\\ \displaystyle 0\\ \displaystyle \end{array} \end{eqnarray*}	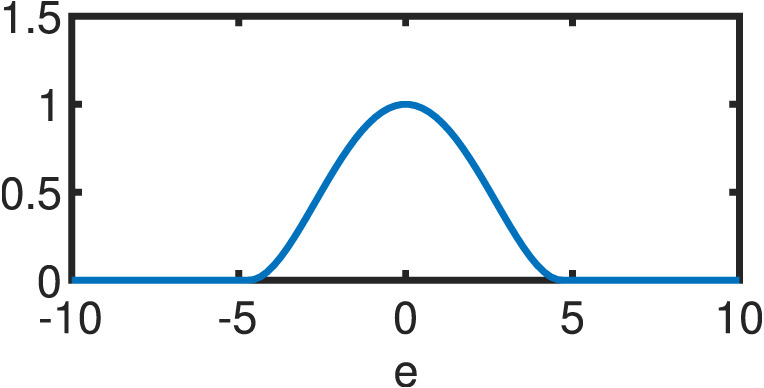
Welsch	$ \frac{{c}^{2}}{2} (1-\exp (-( \frac{e}{c} )^{2}))$	$\exp (-( \frac{e}{c} )^{2})$	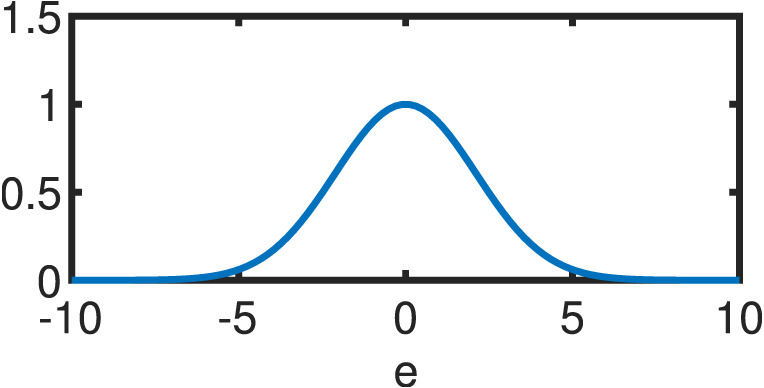

Solving the M-Estimation problem is not as straightforward as the OLS counterpart. In particular, Eq. (20) assumes the optimal **W** is readily available, which, in turn, depends on the residuals, which, again, depends on the coefficient vector. In short, the optimization problem for M–Estimators can be posed as finding both ${\hat {\beta }}_{M-Est}$ and ${\hat {\mathbf{w}}}_{M-Est}$ that minimize Eq. (13). Likewise Half–Quadratic, the common approach is to perform block coordinate descent on the cost function with respect to each variable individually until local optima are found. In the robust regression literature, this optimization procedure is the well known *Iteratively Reweighted Least Squares* or IRLS (Andersen, 2008). The procedure is detailed in Algorithm 1. In particular, the routine runs for either a fixed number of iterations or until the estimates change by less than a selected threshold between iterations. The main hyperparameter is the choice of the robust M–Estimator alongside its corresponding parameter *c*. However, it is conventional to select the value that provides a 95% asymptotic efficiency on the standard Normal distribution (Zhang, 1997). Throughout this work, we exploit such optimal values to avoid parameter tuning by the user (see Table 2). In this way, the influence of outliers and non-Gaussian errors are expected to be diminished in the OMP update stage of the coefficients corresponding to the active set.

**Table 2:** Optimal hyperparameter *c* of M–Estimators according to a 95% asymptotic efficiency on the standard Normal distribution.

Cauchy	Fair	Huber	Tukey	Welsch
2.385	1.4	1.345	4.685	2.985


 
__________________________________________________________________________________________________ 
Algorithm 1 IRLS–based M–Estimation 
__________________________________________________________________________________________________ 
  1:    function IRLS(y ∈ IRm, Φ ∈ IRm × k, wc(u))   ⊳ Weight function w(u) with 
     hyperparameter c 
  2:       t ← 0 
  3:       β(0) = βOLS ← (ΦTΦ)−1ΦTy                          ⊳ OLS initialization 
  4:       e(0) ← y − Φβ(0) 
  5:       MAD ← median|e(0)[i] − ˜ e(0)| 
  6:       s(0) ← 1.4826 × MAD 
  7:       w(0)[i] ← wc(e(0)[i] s(0)  )       i = 1,2,...,m ⊳ Initial weight vector 
  8:       W(0) ← diag(w(0)) 
  9:       t ← 1 
 10:       while NO CONVERGENCE do 
 11:            β(t) ← (ΦTW(t−1)Φ)−1ΦTW(t−1)y       ⊳ Block coordinate descent 
 12:            e(t) ← y − Φβ(t) 
 13:            MAD ← median|e(t)[i] − ˜ e(t)| 
 14:            s(t) ← 1.4826 × MAD 
 15:            w(t)[i] ← wc(e(t)[i] s(t)  )i = 1,2,...,m ⊳ Block coordinatedescent 
 16:            W(t) ← diag( w(t)) 
 17:            t ← t + 1 
 18:       return ˆ βM–Est ← β(t)     ˆ wM–Est ← w(t)                         ⊳ Final estimates    


### M–Estimators–based OMP

Here, we combine the ideas behind greedy approximations to the sparse coding problem and robust M–Estimators; the result is RobOMP or Robust Orthogonal Matching Pursuit. We propose five variants based on five different M–Estimators (Table 1). We refer to each RobOMP alternative according to its underlaying M–Estimator; for instance, Fair–Estimator–based–OMP is simply referred to as *Fair*. As with OMP, the only hyperparameter is the stopping criterion: either *K* as the maximum number of iterations (i.e., sparseness of the solution), or *ϵ*, defined as a threshold on the error norm.

For completeness, Algorithm 2 details the RobOMP routine for the case of set maximum number of iterations (the case involving *ϵ* is straightforward). Three major differences are noted with respect to OMP:

1. The robust M–Estimator–based update stage of the active set is performed via IRLS,
2. The updated residuals are computed considering the weight vector ${\hat {\mathbf{w}}}_{k}$ from IRLS, and
3. The weight vector constitutes an additional output of RobOMP.

The last two differences are key for convergence and interpretability, respectively. The former guarantees shrinkage of the weighted error in its first and second moments, while the latter provides an intuitive, bounded, *m*–dimensional vector capable of discriminating between samples from the main mode and potential outliers at the tails of the density.

 
_______________________________________________________________________________________________________ 
Algorithm 2 RobOMP 
_______________________________________________________________________________________________________ 
  1:  function RobOMP(y ∈ IRm,D ∈ IRm×n,wc(u),K) 
  2:       k ← 1                                                                  ⊳ Initializations 
  3:       r0 ← y 
  4:       Λ0 ←∅ 
  5:       while k < K do 
  6:            λk = argmaxi∈Ω |〈rk−1,di〉|        Ω = {1,2,⋅⋅⋅ ,n} 
  7:            Λk = Λk−1 ∪{λk} 
  8:            Φ = [dΛk[1],dΛk[2],⋅⋅⋅ ,dΛk[k]] 
  9:            {ˆβM–Est, ˆ wk}← IRLS(y,Φ,wc(u))                    ⊳ Robust linear fit 
  10:            xk[Λk[i]] ← ˆ βM–Est[i]                        i = 1,2,...,k       ⊳ Update 
     active set 
  11:            rk[i] ← ˆ wk[i] × (y[i] − (Dxk)[i])                  i = 1,2,...,m        ⊳ 
    Update residual 
  12:            k ← k + 1 
  13:       return xRobOMP ← xK,w ← ˆ wK                           ⊳ Final Estimates    

## Results

This section evaluates the performance of the proposed methods in three different settings. First, sparse coding on synthetic data is evaluated under different noise scenarios. Then, we present an image recognition framework fully–based on sparse decompositions using a well known digital image database. Lastly, a denoising mechanism that exploits local sparse coding highlights the potential of the proposed techniques.

### Sparse coding with synthetic data

The dictionary or observation matrix, **D** ∈ IR^100×500^, is generated with independent entries drawn from a zero–mean Gaussian random variable with variance equal to one. The ideal sparse code, **x**_0_ ∈ IR^500^, is generated by randomly selecting ten entries and assigning them independent samples from a zero–mean, unit–variance Gaussian distribution. The rest of the components are set equal to zero, i.e., *K*_0_ = 10. The resulting observation vector **y** ∈ IR^100^ is computed as the linear combination of the dictionary with weights from the ideal sparse code plus a noise component **n** ∈ IR^100^:

(21) \begin{eqnarray*}\mathbf{y}={\mathbf{Dx}}_{0}+\mathbf{n}.\end{eqnarray*}

The first set of experiments considers different noise distributions. In particular, five noise cases are analyzed: Gaussian ($\mathcal{N}(0,2)$), Laplacian with variance equal to 2, Student’s t–distribution with 2 degrees of freedom, Chi–squared noise with 1 degree of freedom, and Exponential with parameter *λ* = 1. Then, OMP, GOMP, CMP, and the 5 variants of RobOMP estimate the sparse code with parameter *K* = 10. For the active set update stage of CMP and RobOMP, the maximum allowed number of HQ/IRLS iterations is set to 100. For GOMP, *N*_0_ ∈ {2, 3, 4, 5} where the best results are presented.

The performance measure is defined as the normalized ℓ_2_–norm of the difference between the ground truth sparse code, **x**_0_, and its estimate. The average results for 100 independent runs are summarized in Table 3. As expected, most of the algorithms perform similar under Gaussian noise, which highlights the adaptive nature of CMP and RobOMP. For the non–Gaussian cases, CMP and Tukey are major improvements over ordinary OMP. The rest of the RobOMP flavors consistently outperform the state of the art OMP and GOMP techniques. This confirms the optimality of MSE–based greedy sparse decompositions when the errors are Normally distributed; yet, they degrade their performance when such assumption is violated.

**Table 3:** Average norm of sparse code errors of MSE–based OMPs and robust alternatives for different types of noise. Best results are marked bold. *K* = *K*_0_ = 10.

Noise	Gaussian	Laplacian	Student	Chi–squared	Exponential
OMP	5.92	5.69	7.14	5.22	4.43
GOMP	7.66	7.27	9.37	6.71	5.65
CMP	**5.57**	**4.40**	3.87	3.08	**3.49**
Cauchy	5.88	5.21	4.43	3.95	4.06
Fair	5.92	5.34	5.05	4.45	4.13
Huber	5.80	5.04	4.57	3.92	3.89
Tukey	5.85	4.78	**3.80**	**3.05**	3.64
Welsch	5.82	4.84	3.90	3.20	3.70

**Figure 2:**
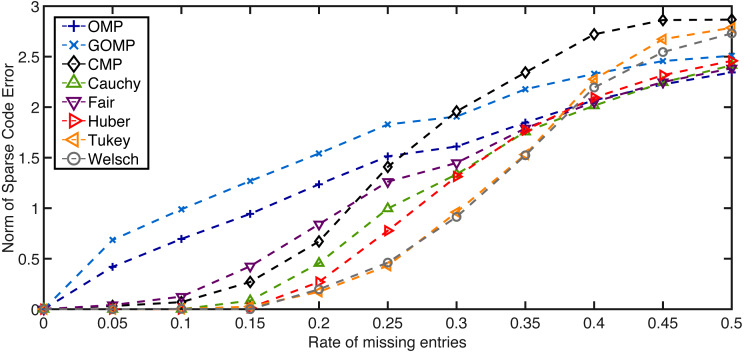
Average normalized norm of sparse code error of MSE–based OMPs and robust alternatives for several rates of missing entries in the observation vector. All algorithms use the ground truth sparsity parameter *K* = *K*_0_ = 10.

The second set of results deals with non–linear additive noise or instance–based degradation. Once again, **D** and **x**_0_ are generated following the same procedure of the previous set of results (*K*_0_ = 10). Yet now, noise is introduced by means of zeroing randomly selected entries in **y**. The number of missing samples is modulated by a rate parameter ranging from 0 to 0.5. Figure 2 summarizes the average results for *K* = 10 and 100 independent runs. As expected, the performance degrades when the rate of missing entries increases. However, the five variants of RobOMP are consistently superior than OMP and GOMP until the 0.4–mark. Beyond that point, some variants degrade at a faster rate. Also, CMP achieves small sparse code error norms for low missing entries rate; however, beyond the 0.25–mark, CMP seems to perform worse than OMP and even GOMP. This experiment highlights the superiority of RobOMP over MSE–based and Correntropy–based methods.

**Figure 3:**
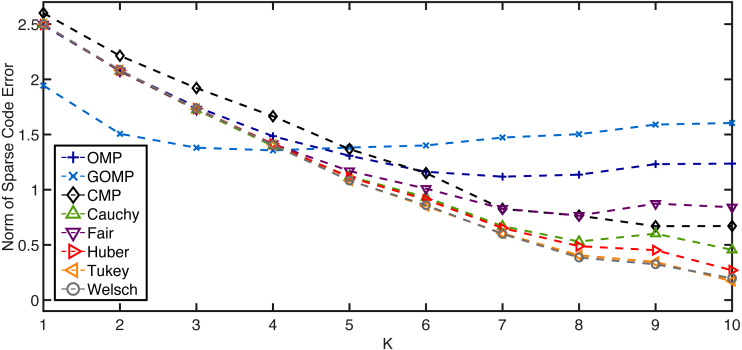
Average normalized norm of sparse code error of MSE–based OMPs and robust alternatives over *K* (number of iterations) for a 0.2 rate of missing entries in the observation vector. *K*_0_ = 10.

Now, the effect of the hyperparameter *K* is studied. Once again, 100 independent runs are averaged to estimate the performance measure. The rate of missing entries is fixed to 0.2 while *K* is the free variable. Figure 3 shows how the average error norm is a non–increasing function of *K* for the non–MSE–based variants of OMP (slight deviation in some cases beyond *K* = 8 might be due to estimation uncertainty and restricted sample size). On the other hand, both OMP and GOMP seem to stabilize after a certain number of iterations, resulting in redundant runs of the algorithm. These outcomes imply that RobOMP is not only a robust sparse code estimator, but also a statistically efficient one that exploits the available information in the data in a principled manner. It is also worth noting that CMP underperforms when compared to most flavors of RobOMP.

**Figure 4:**
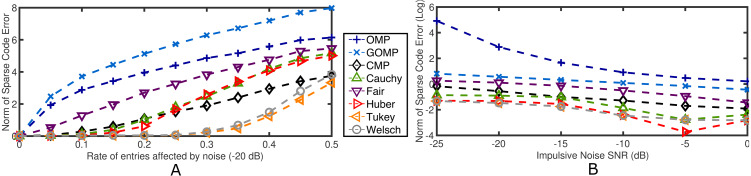
Average normalized norm of sparse code error of MSE–based OMPs and robust alternatives for 2 cases involving impulsive noise in the observation vector. (A) Performance measure for several rates of noisy entries (−20 dB) in the observation vector. (B) Log–Performance measure for several noise levels in the observation vector (fixed 0.10 rate). All algorithms use the ground truth sparsity parameter *K* = *K*_0_ = 10.

Impulsive noise is the other extreme of instance–based contamination. Namely, a rate of entries in **y** are affected by aggressive high–variance noise while the rest of the elements are left intact. The average performance measure of 100 independent runs is reported for *K* = *K*_0_ = 10. Figure 4A details the results for varying rates of entries affected by −20 dB impulsive noise. Again, RobOMP and CMP outperform OMP and GOMP throughout the entire experiment. Tukey and Welsch seem to handle this type of noise more effectively; specifically, the error associated to the algorithms in question seem to be logarithmic or radical for OMP and GOMP, linear for Fair, Cauchy, Huber and CMP, and polynomial for Tukey and Welsch with respect to the percentage of noisy samples. On the other hand, Figure 4B reflects the result of fixing the rate of affected entries to 0.10 and modulating the variance of the impulsive noise in the range [−25,0]. RobOMP and CMP again outperform MSE–based methods (effect visually diminished due to log–transform of the performance measure for plotting purposes). For this case, CMP is only superior to the Fair version of RobOMP.

In summary, the experiments concerning sparse coding with synthetic data confirm the robustness of the proposed RobOMP algorithms. Non–Gaussian errors, missing samples and impulsive noise are handled in a principled scheme by all the RobOMP variants and, for most cases, the results outperform the Correntropy–based CMP. Tukey seems to be the more robust alternative that is able to deal with a wide spectrum of outliers in a consistent, efficient manner.

### RobOMP–based classifier

We introduce a novel robust variant for sparse representation–based classifiers (SRC) fully based on RobOMP. Let ${\mathbf{A}}_{i}=[{\mathbf{a}}_{1}^{i},{\mathbf{a}}_{2}^{i},\ldots ,{\mathbf{a}}_{{n}_{i}}^{i}]\in {\mathrm{IR}}^{m\times {n}_{i}}$ be a matrix with *n*_*i*_ examples from the *i*th class for *i* = 1, 2, …, *N*. Then, denote the set **N** = {1, 2, …, *N*} and the dictionary matrix of all training samples **A** = [**A**_1_, **A**_2_, …, **A**_*N*_] ∈ IR^*m*×*n*^ where $n={\mathop{\sum }\nolimits }_{i=1}^{N}{n}_{i}$ is the number of training examples from all *N* classes. Lastly, for each class i, the characteristic function *δ*_*i*_:IR^*n*^ → IR^*n*^ extracts the coefficients associated with the *i*th label. The goal of the proposed classifier is to assign a class to a test sample **y** ∈ IR^*m*^ given the generative “labeled” dictionary **A**.

The classification scheme proceeds as follows: *N* different sparse codes are estimated via Algorithm 2 given the subdictionaries **A**_*i*_ for *i* = 1, 2, …, *N*. The class–dependent residuals, *r*^*i*^(**y**) are computed and the test example is assigned to the class with minimal residual norm. To avoid biased solutions based on the scale of the data, the columns of **A** are set to have unit–ℓ_2_–norm. The result is a robust sparse representation–based classifier or RSRC, which is detailed in Algorithm 3.

 
_______________________________________________________________________________________________________ 
Algorithm 3 RSRC 
______________________________________________________________________________________________________ 
Inputs: Normalized matrix of training samples A = [A1,A2,...,AN] ∈ IRm×n 
Test Example, y ∈ IRm 
M–Estimator weight function, wc(u) 
Stopping criterion for RobOMP, K 
Output: class(y) 
  1:  (xRobOMP,w) ← RobOMP(y,A,wc(u),K)  ⊳ Compute robust sparse code 
     and weight vector 
  2:  ri(y) = ||diag(w)(y − Aiδi(xRobOMP))||2,   i ∈ N      ⊳ Calculate norm of 
     class–dependent residuals 
  3:  class(y) ← argmini∈N ri(y)                                           ⊳ Predict label    

Similar algorithms can be deployed for OMP, GOMP and CMP (Wang, Tang & Li, 2017). In particular, the original SRC (Wright et al., 2009) exploits a ℓ_1_–minimization approach to the sparse coding problem; however the fidelity term is still MSE, which is sensitive to outliers. In this section we opt for greedy approaches to estimate the sparse representation. Moreover for RobOMP, the major difference is the computation of the residual—we utilize the weight vector to downplay the influence of potential outlier components and, hence, reduce the norm of the errors under the proper dictionary. CMP utilizes a similar approach, but the weight matrix is further modified due to the HQ implementation (see Wang, Tang & Li (2017) for details). We compare the 7 SRC variants under two different types of noise on the Extended Yale B Database.

#### Extended Yale B Database

This dataset contains over 2,000 facial images of 38 subjects under different lighting settings (Lee, Ho & Kriegman, 2005). For each subject, a maximum of 64 frontal–face images are provided alongside light source angles. The original dimensionality of the images is 192 × 168 or 32,256 in vector form. The database can be found at http://vision.ucsd.edu/ iskwak/ExtYaleDatabase/ExtYaleB.html. Due to the difference in lighting conditions, the database is usually segmented into 5 subsets (Wright et al., 2009). Let $\theta =\sqrt{{A}^{2}+{E}^{2}}$ where *A* and *E* are the azimuth and elevation angles of the single light source, respectively. The first subset comprises the interval 0 ≤ *θ* ≤ 12, the second one, 13 ≤ *θ* ≤ 25, the third one, 26 ≤ *θ* ≤ 54, the fourth one, 55 ≤ *θ* ≤ 83, and lastly, the fifth subset includes images with *θ* ≥ 84. In this way, the subsets increase in complexity and variability, making the classifier job more challenging, e.g., subset one includes the cleanest possible examples, while the fifth dataset presents aggressive occlusions in the form of shadows. The cardinality of the five subsets are (per subject): 7, 12, 12, 14, and 19 images. For all the following experiments, the dictionary matrix **A** is built from the samples corresponding to subsets 1 and 2, while the test examples belong to the third subset. This latter collection is further affected by two kinds of non–linearly additive noise.

#### Occlusions and missing pixels

Two different types of noise are simulated: blocks of salt and pepper noise, i.e., occlusions, and random missing pixels. In all the following experiments, the sparsity parameter for learning the sparse code is set to *K* = 5 (for GOMP, *N*_0_ ∈ {2, 3} and the best results are presented). Also, 10 different independent runs are simulated for each noise scenario.

**Figure 5:**
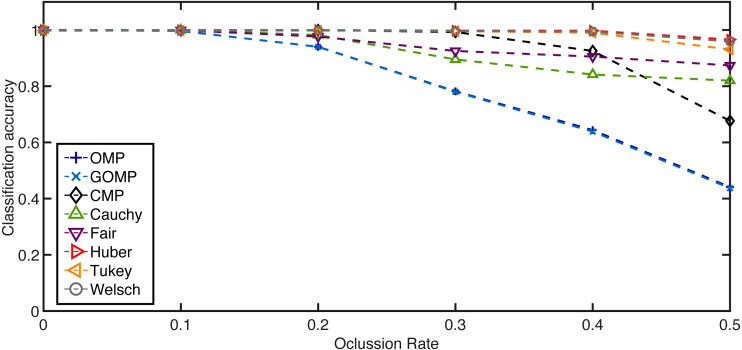
Average classification accuracy on the Extended Yale B Database over occlusion rate of blocks of salt and pepper noise. Feature dimension = 2058. *K* = 5.

For the occlusion blocks, a rate of affected pixels is selected beforehand in the range [0, 0.5]. Then, as in the original SRC (Wright et al., 2009), we downsampled the inputs mainly for computational reasons. In particular, we utilized factors of 1/2, 1/4, 1/8, and 1/16 resulting in feature dimensions of 8232, 2058, 514, and 128, respectively. Next, every test example is affected by blocks of salt and pepper noise (random pixels set to either 0 or 255). The location of the block is random and its size is determined by the rate parameter. Every sample is assigned a label according to SRC variants based on OMP and GOMP, CMP–based classifier (coined as CMPC by Wang, Tang & Li (2017)), and our proposed RSRC. For simplicity, we use the same terminology as before when it comes to the different classifiers. The performance metric is the average classification accuracy in the range [0,1]. Figure 5 highlights the superiority of RSRC over OMP and GOMP. Particularly, Huber, Tukey and Welsch are consistently better than CMP while Fair and Cauchy seem to plateau after the 0.3–mark.

**Figure 6:**
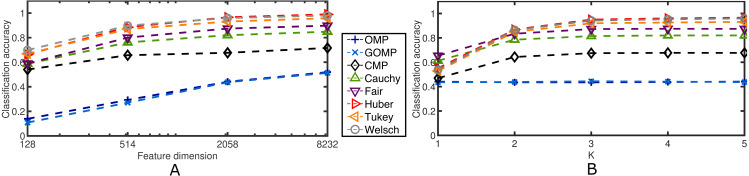
Average classification accuracy on the Extended Yale B Database for two cases concerning blocks of salt and pepper noise at a fixed rate of 0.5. (A) Classification accuracy over feature dimension. *K* = 5. (B) Classification accuracy over sparsity parameter. Feature dimension = 2,058.

Next, the effects of the feature dimension and the sparsity parameter are investigated. Figure 6 confirms the robustness of the proposed discriminative framework. As expected, when the feature dimension increases, the classification accuracy increases accordingly. However, the baselines set by OMP and GOMP are extremely low for some cases. On the other hand, CMP and RSRC outperform both MSE–based approaches, and even more, the novel M–Estimator–based classifiers surpass their Correntropy–based counterpart. When it comes to the sparsity parameter, *K*, it is remarkable how OMP and GOMP do not improve their measures after the first iteration. This is expected due to the lack of principled schemes to deal with outliers. In contrast, RSCR shows a non–decreasing relation between classification accuracy and *K*, which implies progressive refinement of the sparse code over iterations. To make these last two findings more evident, Table 4 illustrates the classification accuracy for a very extreme case: 0.3 rate of occlusion and feature dimension equal to 128, i.e., each input image is roughly 12 × 11 pixels in size (the downsampling operator introduces rounding errors in the final dimensionality). This scenario is very challenging and, yet, most of RSRC variants achieve stability and high classification after only four iterations. On the other hand, OMP and GOMP degrade their performance over iterations. This confirms the robust and sparse nature of the proposed framework.

**Table 4:** Average classification accuracy on the Extended Yale B Database over K for a fixed rate of 0.3 pixels affected by blocks of salt and pepper noise. Best result for each classifier is marked bold. Feature dimension = 128.

K	OMP	GOMP	CMP	Cauchy	Fair	Huber	Tukey	Welsch
1	0.38	**0.36**	0.59	0.53	0.55	0.53	0.51	0.51
2	**0.39**	0.34	0.90	0.81	0.83	0.87	0.86	0.87
3	0.39	0.30	**0.97**	**0.88**	0.88	0.98	0.98	0.98
4	0.37	0.28	0.97	0.88	**0.89**	**0.99**	**0.99**	**0.99**
5	0.36	0.28	0.97	0.88	0.88	0.99	0.99	0.99
6	0.34	0.28	0.97	0.88	0.88	0.99	0.99	0.99
7	0.34	0.28	0.97	0.88	0.88	0.98	0.99	0.99
8	0.33	0.28	0.97	0.88	0.88	0.98	0.99	0.99
9	0.32	0.28	0.97	0.88	0.88	0.98	0.99	0.99
10	0.31	0.28	0.97	0.88	0.88	0.98	0.99	0.98

**Figure 7:**
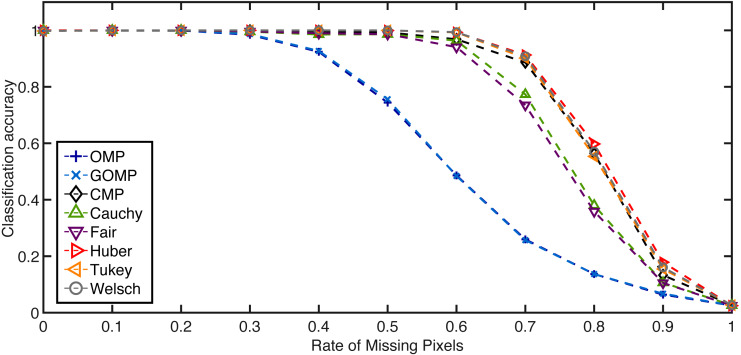
Average classification accuracy on the Extended Yale B Database over missing pixels rate. Feature dimension = 2,058. *K* = 5.

**Figure 8:**
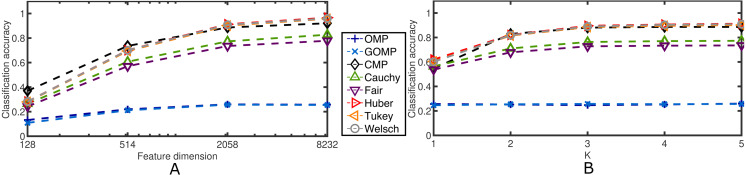
Average classification accuracy on the Extended Yale B Database for two cases concerning missing pixels at a fixed rate of 0.7. (A) Classification accuracy over feature dimension. *K* = 5. (B) Classification accuracy over sparsity parameter. Feature dimension = 2,058.

For the missing pixels case, a rate of affected pixels is selected beforehand in the range [0, 1]. Then, every test example is affected by randomly selected missing pixels—the chosen elements are replaced by samples drawn from a uniform distribution over the range [0, *y*_max_] where *y*_max_ is the largest possible intensity of the image in question. Figures 7 and 8 summarize similar experiments as in the occlusion case. Again, the RSRC are superior than MSE–based methods and consistently increase the performance measure as the sparsity parameter grows. The extreme case here involves a rate of 0.4 affected pixels by distorted inputs and a feature dimension of 128. Table 5 reinforces the notion that robust methods achieve higher classification accuracy even in challenging scenarios.

Lastly, it is worth noting that CMP performs better in the missing pixels case; yet, it fails to surpass the Welsch variant of RSRC which is its equivalent in terms of weight function of errors. Once again, Tukey is the algorithm with overall best results that is able to handle both kinds of noise distributions in a more principled manner.

### Image denoising via robust, sparse and redundant representations

The last set of results introduces a preliminary analysis of image denoising exploiting sparse and redundant representations over overcomplete dictionaries. The approach is based on the seminal paper by Elad & Aharon (2006). Essentially, zero–mean white and homogeneous Gaussian additive noise with variance *σ*^2^ is removed from a given image via sparse modeling. A global image prior that imposes sparsity over patches in every location of the image simplifies the sparse modeling framework and facilitates its implementation via parallel processing. In particular, if the unknown image **Z** can be devised as the spatial (and possibly overlapping) superposition of patches that can be effectively sparsely represented given a dictionary **D**, then, the optimal sparse code, ${\hat {\mathbf{x}}}_{ij}$, and estimated denoised image, $\hat {\mathbf{Z}}$, are equal to:

(22) \begin{eqnarray*}\{{\hat {\mathbf{x}}}_{ij},\hat {\mathbf{Z}}\}=\argmin _{{\mathbf{x}}_{ij},\mathbf{Z}}\lambda {|}{|}\mathbf{Z}-\mathbf{Y }{|}{\mathop{{|}\nolimits }\nolimits }_{2}^{2}+\sum _{ij}{\mu }_{ij}{|}{|}{\mathbf{x}}_{ij}{|}{{|}}_{0}+\sum _{ij}{|}{|}\mathbf{D}{\mathbf{x}}_{ij}-{\mathbf{R}}_{ij}\mathbf{Z}{|}{\mathop{{|}\nolimits }\nolimits }_{2}^{2}\end{eqnarray*}

where the first term is the log–likelihood component that enforces close resemblance (or proximity in an ℓ_2_ sense) between the measured noisy image, **Y**, and its denoised (and unknown) counterpart **Z**. The second and third terms are image priors that enforce that every patch, **z**_*ij*_ = **R**_*ij*_**Z**, of size $\sqrt{n}\times \sqrt{n}$ in every location of the constructed image **Z** has a sparse representation with bounded error. *λ* and *μ*_*ij*_ are regularization parameters than can easily be reformulated as constraints.

**Table 5:** Average classification accuracy on the Extended Yale B Database over K for a fixed rate of 0.4 missing pixels. Best result for each classifier is marked bold. Feature dimension = 128.

K	OMP	GOMP	CMP	Cauchy	Fair	Huber	Tukey	Welsch
1	0.51	**0.54**	0.57	0.61	0.62	0.56	0.54	0.54
2	0.54	0.52	0.89	0.87	0.86	0.88	0.88	0.88
3	**0.57**	0.48	**0.95**	0.91	**0.90**	0.93	0.94	0.94
4	0.56	0.45	0.95	**0.92**	0.90	**0.94**	0.96	0.95
5	0.55	0.45	0.95	0.92	0.89	0.94	0.96	**0.96**
6	0.54	0.45	0.95	0.91	0.89	0.94	**0.97**	0.96
7	0.53	0.45	0.94	0.91	0.89	0.94	0.97	0.96
8	0.52	0.45	0.94	0.91	0.89	0.94	0.96	0.96
9	0.51	0.45	0.94	0.91	0.89	0.94	0.96	0.95
10	0.50	0.45	0.94	0.91	0.89	0.93	0.96	0.95

Block coordinate descent is exploited to solve Eq. (22). In particular, ${\hat {\mathbf{x}}}_{ij}$ is estimated via greedy approximations of the sparse code of each local block or patch. The authors suggest OMP with stopping criterion set by ${|}{|}\mathbf{D}{\mathbf{x}}_{ij}-{\mathbf{R}}_{ij}\mathbf{Z}{|}{\mathop{{|}}\nolimits }_{2}^{2}\leq (C\sigma )^{2}$ for all {*ij*} combinations (sequential sweep of the image to extract all possible $\sqrt{n}\times \sqrt{n}$ blocks). Then, the estimated denoised image has the following closed form solution:

(23) \begin{eqnarray*}\hat {\mathbf{Z}}= \left( \right. \lambda I+\sum _{ij}{\mathbf{R}}_{ij}^{T}{\mathbf{R}}_{ij}{ \left( \right. }^{-1} \left( \right. \lambda \mathbf{Y }+\sum _{ij}{\mathbf{R}}_{ij}^{T}\mathbf{D}{\mathbf{x}}_{ij} \left( \right. \end{eqnarray*}

where *I* is the identity matrix. The authors go one step further and propose learning the dictionary, **D**, as well; this is accomplished either from a corpus of high–quality images or the corrupted image itself. The latter alternative results in a fully generative sparse modeling scheme. For more details regarding the denoising mechanisms, refer to Elad & Aharon (2006).

For our case, we focus on the sparse coding subproblem alone and utilize an overcomplete Discrete Cosine Transform (DCT) dictionary, **D** ∈ IR^64×256^, and overlapping blocks of size 8 × 8. The rest of the free parameters are set according to the heuristics presented in the original work: *λ* = 30∕*σ* and *C* = 1.15. Our major contribution is the robust estimation of the sparse codes via RobOMP in order to handle potential outliers in a principled manner. Two types of zero–mean, homogeneous, additive noise (Gaussian and Laplacian) are simulated with different variance levels on 10 independent runs. Each run comprises of separate contaminations of 4 well known images (Lena, Barbara, Boats and House) followed by the 7 different denoising frameworks, each one based on a distinct variant of OMP. As before, every algorithm is referred to as the estimator exploited in the active set update stage.

Tables 6 and 7 summarize the average performance measures (PSNR in dB) for 5 different variance levels of each noise distribution. As expected, OMP is roughly the best denoising framework for additive Gaussian noise. However, in the Laplacian case, Cauchy achieves higher PSNR levels throughout the entire experiment. This suggests the Cauchy M–Estimator is more suitable for this type of non–Gaussian environment. It is worth noting though that the averaging performed in Eq. (23) could easily blur the impact of the sparse code solvers for this particular joint optimization. Also, no attempt was made to search over the hyperparameter space of *λ* and *C*, which we suspect have different empirical optima depending on the noise distribution and sparse code estimator. These results are simply preliminary and highlight the potential of robust denoising frameworks based on sparse and redundant representations.

**Table 6:** Grand average PSNR (dB) of estimated denoised images under zero-mean additive Gaussian noise exploiting patch-based sparse and redundant representations.

*σ*	OMP	GOMP	CMP	Cauchy	Fair	Huber	Tukey	Welsch
5	36.33	36.31	35.62	**36.56**	36.55	36.52	36.20	36.29
10	32.38	32.36	31.01	**32.44**	32.22	32.39	32.17	32.17
15	**30.35**	30.33	28.95	30.25	29.88	30.21	30.01	29.97
20	**28.97**	28.96	27.85	28.78	28.40	28.76	28.58	28.53
25	**27.93**	27.92	27.12	27.70	27.39	27.70	27.55	27.51

**Table 7:** Grand average PSNR (dB) of estimated denoised images under zero-mean additive Laplacian noise exploiting patch-based sparse and redundant representations.

*σ*	OMP	GOMP	CMP	Cauchy	Fair	Huber	Tukey	Welsch
5	36.27	36.25	35.64	**36.59**	36.56	36.56	36.21	36.30
10	32.22	32.19	31.03	**32.44**	32.20	32.38	32.15	32.15
15	30.09	30.05	28.97	**30.20**	29.83	30.15	29.95	29.90
20	28.63	28.58	27.88	**28.70**	28.33	28.66	28.50	28.45
25	27.51	27.45	27.14	**27.60**	27.30	27.58	27.45	27.41

## Discussion

An example is considered a univariate outlier if it deviates from the rest of the distribution for a particular variable or component (Andersen, 2008). A multivariate outlier extends this definition to more than one dimension. However, a regression outlier is a very distinctive type of outlier—it is a point that deviates from the linear relation followed by most of the data given a set of predictors or explanatory variables. In this regard, the current work focuses on regression outliers alone. The active set update stage of OMP explicitly models the interactions between the observation vector and the active atoms of the dictionary as purely linear. This relation is the main rationale behind RobOMP: regression outliers can be detected and weighted when M–Estimators replace the pervasive OLS solver. If the inference process in sparse modeling incorporates higher–order interactions (as in Vincent & Bengio (2002)), linear regression outliers become meaningless and other techniques are needed to downplay their influence. The relation between outliers in the observation vector and regression outliers is highly complex due to the mixing of sources during the generative step and demands for further research.

Even though other OMP variants are utilized in practice for different purposes, e.g., GOMP, ROMP and CoSaMP, we decided to disregard the last two flavors mainly due to three factors: space limitations, inherent MSE cost functions, and most importantly, they both have been outperformed by CMP in similar experiments as the ones simulated here (Wang, Tang & Li, 2017). The algorithm to beat was CMP due to its resemblance to an M–Estimator–based OMP. We believe we have provided sufficient evidence to deem RobOMP (and specifically the Tukey variant) as superior than CMP in a wide variety of tasks, performance measures and datasets. In this regard, it is worth noting that CMP reduces to the Welsch algorithm with the ℓ_2_–norm of the errors as the estimated scale parameter (*s* = ||**e**||_2_), and hyperparameter $c=\sqrt{m}$. The main drawback of such heuristic is the use of a non–robust estimator of the scale, which in turn, will bias the sparse code. The CMP authors introduce a data–dependent parameter of the exponential weight function (Gaussian kernel of Correntropy) that relies on the dimensionality of the input, *m*. The rationale behind such add–hoc choice is not fully justified, while in contrast, we provide statistically sound arguments for our choice of the weight function hyperparameter, i.e., 95% asymptotic efficiency on the standard Normal distribution. We believe this is the underlying reason behind the superiority of Welsch over CMP on most of the synthetic data experiments and the entirety of the simulations on the Extended Yale B Database.

M–Estimators are not the only alternative to robust linear regression. S–Estimators (Rousseeuw & Yohai, 1984) are based on the residual scale of M–Estimators. Namely, S–estimation exploits the residual standard deviation of the errors to overcome the weakness of the re–scaled MAD. Another option is the so–called MM–Estimators (Yohai, 1987) which fuse S–Estimation and M–Estimation to achieve high breakdown points (BDP) and better efficiency. Optimization for both S–Estimators and MM–Estimators is usually performed via IRLS. Another common approach is the Least Median of Squares method (Rousseeuw, 1984) where the optimal parameters solve a non–linear minimization problem involving the median of squared residuals. Advantages include robustness to false matches and outliers, while the main drawback is the need for Monte Carlo sampling techniques to solve the optimization. These three approaches are left for potential further work in order to analyze and compare performances of several types of robust estimators applied to sparse coding.

In terms of image denoising via robust, sparse and redundant representations, future work will involve the use of the weight vector in the block coordinate descent minimization in order to mitigate the effect of outliers. If sparse modeling is the final goal, K–SVD (Aharon, Elad & Bruckstein, 2006) is usually the preferred dictionary learning algorithm. However, in the presence of non–Gaussian additive noise, the estimated dictionary might be biased as well due to the explicit MSE cost function of the sequential estimation of generative atoms. Plausible alternatives include Correntropy–based cost functions (Loza & Principe, 2016) and ℓ_1_–norm fidelity terms (Loza, 2018).

In the spirit of openness and to encourage reproducibility, the MATLAB (Mathworks) and Python code corresponding to all the proposed methods and experiments of this paper are freely available at https://github.com/carlosloza/RobOMP.

## Conclusion

We investigated the prospect of M–estimation applied to sparse coding as an alternative to LS–based estimation and found that our hypothesis of exploiting M–estimators for better robustness is indeed valid. Unlike the original Orthogonal Matching Pursuit, our framework is able to handle outliers and non–Gaussian errors in a principled manner. In addition, we introduce a novel robust sparse representation–based classifier that outperform current state of the art and similar robust variants. Preliminary results on image denoising confirm the plausibility of the methods and open the door to future applications where robustness and sparseness are advantageous. The proposed five algorithms do not require parameter tuning from the user and, hence, constitute a suitable alternative to ordinary OMP.

## Additional Information and Declarations

### Data Availability

The following information was supplied regarding data availability: Code is available at GitHub:
https://github.com/carlosloza/RobOMP
Data (Extended Yale B Database) is available at:
http://vision.ucsd.edu/ iskwak/ExtYaleDatabase/ExtYaleB.html.
